# Insight into the inhibitory activity of mangiferin and Silybin against HER2 and EGFR using theoretical and experimental approaches

**DOI:** 10.1038/s41598-025-93612-2

**Published:** 2025-03-13

**Authors:** Jesús Eduardo Alvarado-Lozano, Jorge Arturo Hernández-Valencia, Rodolfo Daniel Ávila-Avilés, Martiniano Bello

**Affiliations:** 1 Laboratorio de Diseño y Desarrollo de Nuevos Fármacos e Innovación Biotecnológica, Sección de Estudios de Posgrado e Investigación, Escuela Superior de Medicina, Instituto Politécnico Nacional, Plan de San Luis y Salvador Diaz Mirón s/n, Casco de Santo Tomás, Miguel Hidalgo, Mexico City, 11340 México; 2 Transdisciplinary Research for Drug Discovery, Sociedad Mexicana de Epigenética y Medicina Regenerativa A. C. (SMEYMER), Mexico City, México; 3 Centro Conjunto de Investigación en Química Sustentable (CCIQS), UAEM-UNAM, Toluca, Estado de México 50200 México

**Keywords:** HER2, EGFR, Silybin, Mangiferin Docking, MD simulations, Breast cancer, Structure-based drug design, Computational biology and bioinformatics

## Abstract

Despite the advances made in diagnosing and treating breast cancer, it continues to pose a significant threat to women’s health. High-risk mutations can lead to high resistance to current treatments and poor prognosis. Therefore, new treatment strategies are needed. Mangiferin and silybin, two natural substances obtained from plants, have demonstrated encouraging results as anticancer drugs. This study investigated the activity of these compounds against two therapeutic targets, human epidermal growth factor receptor 2 (HER2) and epidermal growth factor receptor (EGFR). We assessed the binding affinity and stability of these compounds with the active sites of wild-type and mutated HER2 and EGFR by using computational screening techniques, namely molecular docking, density functional theory, and molecular dynamics (MD) simulations with the MMGBSA method. We used molecular docking, triplicate MD simulations summing 300 ns each, and density functional theory analysis to estimate the binding mechanism of mangiferin and silybin inside the wild-type and mutated EGFR and HER2 active regions. Moreover, an in vitro experiment showed that mangiferin and silybin inhibited the growth of two HER2-positive breast cancer cell lines, BT-474 and SK-BR-3, at micromolar concentrations. These findings suggest the potential for developing novel anticancer therapies that specifically target EGFR and HER2.

**Supplementary Information:**

The online version contains supplementary material available at 10.1038/s41598-025-93612-2.

## Introduction

Four tyrosine kinase receptors make up the human epidermal growth factor receptor (EGFR) family: human epidermal growth factor receptor 1 (HER1 or EGFR), 2 (HER2), 3 (HER3), and 4 (HER4)^1^. These receptors have four different regions: an extracellular domain, a hydrophobic transmembrane segment, an intracellular region containing a juxtamembrane region, and a protein kinase domain, which is one of the most studied domains in the development of anticancer medications^2^. Physiological activation of EGFRs occurs when endogenous growth factors bind to their extracellular domains. This encourages the formation of asymmetric homo- and heterodimers between the four human EGFR members, with HER2 serving as the primary component of EGFR heterodimers^3^. Dimer formation results in the movement of the N-terminal αC helix and a conformational change in the activation loop of protein kinase domain, the DFG motif, forming the active state^4^. Certain areas crucial in the catalytic process, such as the hinge, P-loop, DFG motif, and activation loop, are shared by the protein kinase domains of these enzymes (Fig. S1). The active or inactive states of the enzymes are also related to their catalytic activity. The activation loop is in the DFG-in conformation when it is active and in the DFG-out conformation when it is inactive. The catalytic binding site is blocked in the inactive state by the Asp residue in the DFG motif, which aids in the transfer of the phosphate group from adenosine triphosphate (ATP) to the substrate. In the active state, however, a conformational change in the activation loop permits access to the ATP binding site^5^.

Previously reported structural data have provided insights into the molecular recognition between a ligand and EGFR or HER2 in the active and inactive states^6,7^. In this context, targeting the inactive conformation of EGFR has been reported to yield highly specific ligands; however, targeting the active conformation is only likely to be beneficial in diseases resulting from mutations that promote the activate state, as these inhibitors are promiscuous, meaning they have the ability to target multiple protein kinases^8^.

Enzyme phosphorylation has a role in altering the physicochemical conditions of the active site of the enzyme, which permits protein kinases to evolve into their fundamental roles. On the other hand, certain conditions, such variations in protein kinase expression levels or mutations in their ATP binding sites, influence cellular events by altering the catalytic activity or creating a constitutively active protein kinase. Mutations at the gatekeeper, activation loop location, and glycine-rich loop impact the catalytic activity of the ATP binding site. For EGFR, three examples of mutations that destabilize the inactive conformation are G719S, which is proximal to the glycine-rich loop, L858R in the activation loop, and T790M at the gatekeeper position^9^. T790M prevents access to the catalytic binding site in EGFR, which has bulkier side chain replacements. This mutation affects the hydrogen bonds between threonine and the ligand, encouraging the constitutive active state of EGFR but without affecting the ATP binding site. T798M substitution in HER2 is analogous to the gatekeeper T790M substitution in EGFR^10^, conferring strong resistance to lapatinib. Constitutive activation of both EGFR and HER2 is caused by mutations and results in resistance to radiation and chemotherapy that is associated with the advancement of various cancer types, including lung and breast malignancies^11^.

Drug kinase inhibitors approved by the Food and Drug Administration (FDA) are classified into two categories: those that target the active or inactive forms of EGFR. Among these, lapatinib and gefitinib are dual inhibitors of EGFR and HER2 in their inactive states^2,12^. These competitive inhibitors of ATP have been approved to treat breast, lung, colorectal, and pancreatic cancers. Nevertheless, using these compounds is linked to medication resistance^13^. Therefore, there is a need to develop novel EGFR and HER2 inhibitors.

Mangiferin and silybin, two plant-derived natural compounds , have shown promising anticancer activity ^14–17^. In addition, a recent computational study showed that mangiferin binds to wild-type HER2 with high affinity^18^. On the other hand, there is no structural information about the binding of silybin to wild-type or mutant HER2, or whether silybin and mangiferin bind to wild-type or mutant EGFR. Thus, in the present study, we explored the binding of mangiferin and silybin to wild type EGFR and HER2 and as well as to EGFR and HER2 carrying mutations that impact ligand stabilization. We evaluated the maintenance of the predicted docking for the wild-type and mutant receptor–ligand complexes using triplicate molecular dynamics simulations, each combined with the molecular mechanics generalized Born surface area approach (MMGBSA). Moreover, we applied density functional theory (DFT) to determine the reactivity of the compounds at the ATP binding site. Finally, we validated the predicted inhibitory properties using two HER2-positive human breast cancer cell lines, BT-474 and SK-BR-3, and identified experimental affinities at micromolar concentrations. This study provides the first structural and functional analysis of silybin and mangiferin as potential inhibitors of HER2 and EGFR, including their mutated forms. It fills a knowledge gap by evaluating their interactions with drug-resistant mutants and testing them in HER2-positive breast cancer cell lines. These findings support the further optimization and development of these natural compounds as alternative cancer therapies targeting HER2 and EGFR.

## Methods

### Preparation of systems

The structures of silybin and mangiferin were obtained from ChemSpider http://www.chemspider.com/. These compounds were optimized at the AM1 level with the MOE2022 software https://www.chemcomp.com/en/Products.htm^19^. The wild-type and mutant EGFR and HER2 models were obtained from the PDB to form the protein–ligand complexes: human wild-type EGFR (PDB entry 3W2S; Resolution 1.90 Å; Chain A), L858R EGFR mutant (PDB entry ITZ, Resolution 2.80 Å; Chain A), L858R/T790M EGFR mutant (PDB entry 3W2R; Resolution 2.05 Å; Chain A), G719S EGFR mutant (PDB entry 2ITO; Resolution 2.90 Å; Chain A), and HER2 (PDB entry 7JXH; Resolution 3.27 Å; Chain A). The T798M HER2 mutant was constructed using the mutagenesis wizard in the PyMol 0.99rc6. software http://www.pymol.org^20^ and PDB entry 7JXH. Other chains, water molecules, and unnecessary heteroatoms were removed. Protein protonation states were assigned at pH 7.4 to reflect physiological conditions. Asp, Glu, His, Lys, and Arg side chains were checked for correct protonation states.

### Molecular docking

SwissDock^21^ was used for molecular docking, using the standard settings. The shapes of the ligands were optimized with Avogadro^22^, employing a UFF force field and the steepest descent approach, which was then enhanced with conjugate gradient algorithms. In each scenario, the search area was established using blind docking, and the receptor–ligand complex with the lowest binding score was selected as the representative complex. The following details summarize the docking protocol used: The entire protein surface was selected as the docking search area. A grid-based docking algorithm was used to evaluate multiple binding sites. Ligands were considered flexible with rotatable bonds allowed to explore different conformations. The receptor (EGFR/HER2) was kept rigid, meaning side chains did not move during docking. SwissDock generates multiple docking poses (clusters) and ranks them based on binding energy scores. The CHARMM22 force field was used to calculate interaction energies. The most favorable binding conformations were selected based on: Binding free energy (ΔG), and Clustering of docking poses in energetically favorable regions. gefitinib was selected as a positive control because there are experimental studies confirming its binding to the evaluated receptors.

### Molecular dynamics simulations

Before running the molecular dynamics simulations, the systems were minimized and equilibrated. The minimization involved 5000 iterations of steepest descent and 4000 iterations of conjugate gradient minimization. The temperature of the systems was raised from 0 to 310 K for 200 ps while maintaining an NVT ensemble, utilizing a Berendsen thermostat^23^. During this process, the heavy atoms in the protein were limited by an elastic constant of 3 kcal/mol/Å^2^. Subsequently, the density was equilibrated for 200 ps within an NTP ensemble, utilizing a Langevin thermostat^24,25^ and a Berendsen barostat^23^. Subsequently, a constant pressure was maintained for 600 ps to achieve equilibration at 310 K, while preserving the same constraints on the heavy atoms that were applied during the heating phase. Molecular dynamics simulations were conducted as described above, without imposing any constraints. The van der Waals forces and short-range electrostatic interactions were defined with a cutoff of 10 Å, while the long-range electrostatic interactions were addressed using the particle mesh Ewald (PME) method^26^. The simulations utilized a time step of 2 fs, and the SHAKE algorithm^27^ was implemented to maintain the bond lengths between hydrogen atoms and their associated heavy atoms. The molecular dynamics simulations were conducted in triplicate by utilizing the pmemd.cuda module within Amber22 https://ambermd.org/AmberMD.php^28^. A unified trajectory was generated by merging the three simulations, which was then used to calculate the root-mean-square deviation (RMSD), the radius of gyration (Rg), the root-mean squared fluctuation (RMSF) and to perform clustering analysis as well as binding free energy (ΔG_bind_) assessments. Figures were generated using PyMol^20^ software.

### ΔGbind and per-residue decomposition calculations

ΔG_bind_ for each system was calculated over the final 20 ns of the triplicate 100 ns molecular dynamics trajectories. The analysis was performed using 200 snapshots obtained at time intervals of 100 ps from the last 20 ns of MD simulations. This was accomplished using the MMGBSA technique, along with the single-method molecular dynamics simulation protocol utilizing the MMPBSA.py module^29^ available in the Amber22 simulation software^28^. These methodologies allow for the decomposition of ΔG_bind_ into its energetic components, as detailed previously^30^.

### DFT analysis

The structures of silybin, mangiferin, and gefitinib were obtained from the PubChem database using CID numbers 31553, 5281647, and 123631, respectively. The electronic properties were evaluated with DFT to determine the most stable configurations and to obtain molecular descriptors, including the molecular electrostatic potential (MEP), the frontier molecular orbitals (FMOs, including the highest occupied molecular orbital [HOMO] and the lowest unoccupied molecular orbital [LUMO], and global reactivity descriptors (electron affinity, ionization energy, electronegativity, electrophilicity indices, global hardness and softness, and chemical potential). The Gaussian 09: https://gaussian.com^31^ was used to perform DFT calculations, employing the B3LYP functional model^32^ and the polarized 6-311 + + G(2d,2p) basis set. To confirm vibrational frequencies and to ensure they correspond to the global minimum of the potential energy surface, all vibrational analyses were evaluated. Visualization of the structures and orbitals was completed using the VESTA 3.4.5 https://jp-minerals.org/vesta/en/download.html^33^ and Molden software https://www.theochem.ru.nl/molden/^34^. Global reactivity descriptors were calculated using the TAFF 1.1 software https://github.com/HumanOsv/TAF^35^.

### Cell lines and cell culture

Two HER2-positive human breast cancer cell lines, BT-474 and SK-BR-3, were obtained from ATCC and cultured in Dulbecco’s Modified Eagle’s Medium (DMEM) supplemented with 10% (v/v) fetal bovine serum, penicillin (100 U/mL), streptomycin (100 µg/mL), and amphotericin B (0.25 µg/mL) in a 5% CO_2_ incubator at 37 °C. Mangiferin, silybin, and gefitinib stock solutions were prepared in dimethyl sulfoxide (DMSO) at a concentration of 2.5 mM. The compounds were diluted in culture medium to the final concentration indicated for each experiment.

### Cell viability assay

Cells were plated at a density of 3 × 10^4^ cells/well in 96-well plates 24 h before the assay. Then, they were treated for 48 h in a dose-dependent manner: 25, 50, 75, 100, 150 and 200 µM for mangiferin or silybin, and gefitinib was used as a market drug with 0.5, 1, 2.5, 5, 10 and 15 µM. Then, they were treated with the indicated doses of the compounds for 48 h. At the end of the treatment period, the cells were incubated with 3-(4,5-dimethylthiazol-2-yl)-2,5-diphenyltetrazolium bromide (MTT, 0.5 mg/mL) for 45 min. The medium was removed, the synthesized formazan dye crystals were solubilized with 200 µL of acid isopropanol, and absorbance was measured at 570 nm. The growth percentage was calculated considered the number of control cells with vehicle as 100%.

### Statistical analysis

The results are expressed as the mean ± standard deviation of at least three independent experiments. The half-maximal inhibitory concentration (IC_50_) for each compound was determined using nonlinear regression (curve fit) based on the log [inhibitor] versus normalized response–variable slope method in GraphPad Prism version 8.0.2 for Windows, GraphPad Software, Boston, Massachusetts USA, www.graphpad.com.

## Results

### Stability of the simulated systems

The mobility of the receptor–ligand complexes is displayed in the RMSD and Rg graphs (Figs. S2 and S3). The Rg values oscillated between 19.6 and 20.8 Å (Fig. S3). Overall, the receptor–ligand complexes displayed RMSD (Fig. S2) and Rg (Fig. S3) oscillations that showed constant behavior between 40 and 80 ns. Thus, for the subsequent analysis we eliminated the first 80 ns.

### Root mean square fluctuation (RMSF) analysis

RMSF analysis is a key indicator of structural flexibility in molecular dynamics simulations, allowing the identification of highly mobile regions within receptor-ligand complexes. In this study, RMSF analysis was performed after excluding the first 80 ns of the 100-ns MD simulations to ensure that calculations reflected an equilibrated state of the evaluated structures (Fig. S4).

RMSF analysis demonstrated that the highest mobility regions in wild-type and mutant EGFR-ligand systems was localized at N-terminal region, followed by the loop connecting β-sheet 7 and α-helix 4 (858–888), the loop between α-helices 5 and 6 to the loop between α-helices 6 and 7 (910–940), the loop between α-helices 7 and C-terminal region (979–1011), loop connecting β-sheet 1 and 2 (721–723), loop connecting β-sheet 2 and 3 (729–742), and loop connecting β-sheet 3 and α-helix 1 (747–752) (Fig. S4A). The EGFR–ligand systems showed the highest mobility at the loop connecting β-sheet 7 and α-helix 4 (858–888), the loop between α-helices 5 and 6 to the loop between α-helices 6 and 7 (910–940), and the loop between α-helices 7 and C-terminal region (979–1011) (Fig. S4A) compared with EGFR_L858R−ligand_ (Fig. S4B), EGFR_T790M−L858R−ligand_ (Fig. S4C) or EGFR_G719S−ligand_ (Fig. S4D) systems, however, EGFR_T790M−L858R−ligand_ systems showed the highest mobility at the loop connecting β-sheet 7 and α-helix 4 (858–888), and the loop connecting β-sheet 3 and α-helix 1 (747–752).

In wild-type and mutant HER2_− ligand_ systems the highest mobility was localized at C-terminal region, followed by the loop connecting β-sheet 7 and α-helix 4 (862–876), loop connecting β-sheet 1 and 2 (729–731), loop connecting β-sheet 2 and 3 (740–747), and loop connecting β-sheet 3 and α-helix 1 (756–759). The HER2_–ligand_ systems (Fig. S4E) showed higher mobility at the loop connecting β-sheet 7 and α-helix 4 (862–876) compared with HER2_T798M − ligand_ (Fig. S4F).

### Molecular dynamics simulations of EGFR–ligand interactions

**Fig. 1:**
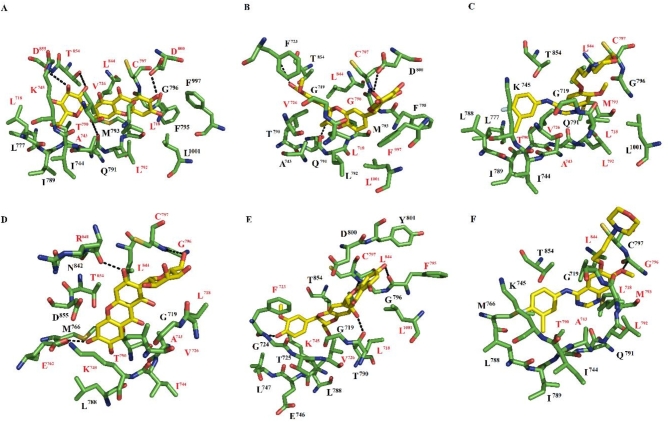
The receptor–ligand conformations for EGFR–mangiferin (**A**), EGFR–silybin (**B**), EGFR–gefitinib (**C**), EGFR_L858R_–mangiferin (**D**) EGFR_L858R_–silybin (**E**), and EGFR_L858R_–gefitinib (**F**).

The interactions between EGFR and mangiferin, silybin, or gefitinib were stabilized by approximately 18–19 amino acid residues (Fig. 1). Among these residues, Leu718, Val726, Ala743, Lys745, Thr790, Gln791, Leu792, Met793, Gly796, Cys797, Leu844, Thr854, and Leu1001 were consistently present in most of the complexes. Additionally, there was hydrogen bonding in certain complexes. Specifically, mangiferin formed hydrogen bonds with the side chain atoms of Thr790, Gln791, Asp800, Thr854, and Asp855 (Fig. 1A). Silybin established hydrogen bonds with the side chain atoms of Asp800 and Asp855, as well as the backbone atoms of Gly791 and Cys797 (Fig. 1B). However, gefitinib did not form any hydrogen bonds (Fig. 1C).

The interactions between EGFR_L858R_ and mangiferin, silybin, or gefitinib were supported by 16–19 residues (Table S1). Among these amino acid residues, we identified Leu718, Gly719, Ala743, Lys745, Leu788, Thr790, Gly796, Cys797, Leu844, and Thr854 in all three complexes. Mangiferin established hydrogen bonds with the side chain atoms of Glu762, and with the backbone atoms of Arg841 and Cys797 (Fig. 1D). Silybin formed three hydrogen bonds with the backbone atoms of Phe723, Leu718, Cys797, and Phe795 (Fig. 1E). Finally, gefitinib formed a single hydrogen bond with a backbone atom of Gly796 (Fig. 1F).

**Fig. 2:**
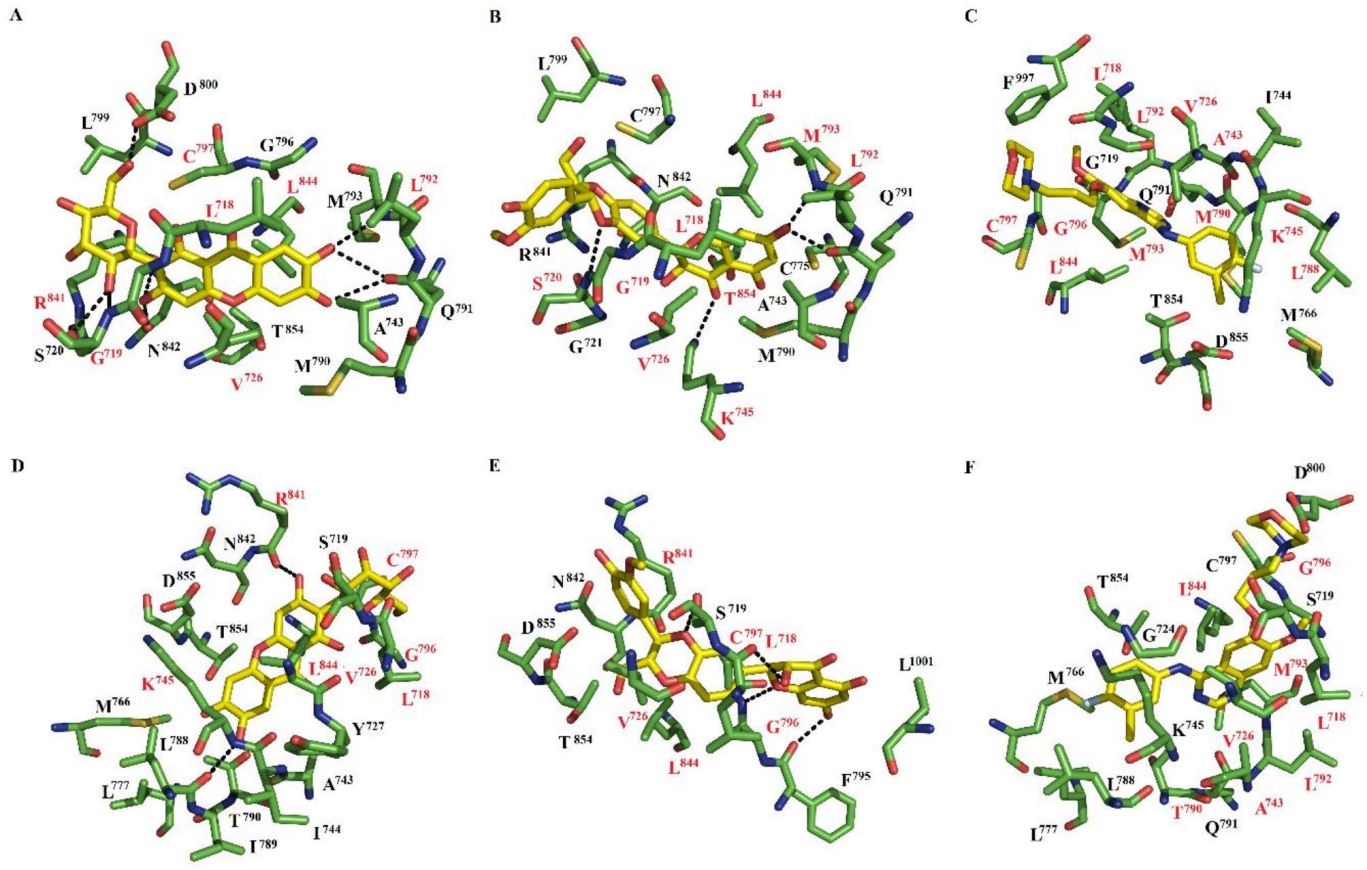
The receptor–ligand conformations for EGFR_T790M−L858R_–mangiferin (**A**), EGFR_T790M−L858R_–silybin (**B**), EGFR_T790M−L858R_–gefitinib (**C**), EGFR_G719S_–mangiferin (**D**) EGFR_G719S_–silybin (**E**), and EGFR_G719S_–gefitinib (**F**).

The interactions between mangiferin, silybin, and gefitinib and EGFR_T790M−L858R_ were supported by 16–18 residues (Table S1). The three complexes shared 11 amino acid residues, including Leu718, Gly719, Val726, Ala743, Met790, Gln791, Leu792, Met793, Cys797, Leu844, and Thr854. Mangiferin established two hydrogen bonds with the side chain atom of Asp800 and Asn842, along with six hydrogen bonds with the backbone atoms of Gln791, Met793, Ser720, and Arg841 (Fig. 2A). Silybin engaged in one hydrogen bond with the side chain atom of Lys745, and three hydrogen bonds with backbone atoms of Ser720, Gln721, and Met793 (Fig. 2B). On the other hand, gefitinib did not form hydrogen bonds (Fig. 2C).

The interactions between EGFR_G719S_ and mangiferin, silybin, or gefitinib involved 12–18 residues (Table S1). There were seven common amino acid residues in all three complexes: Leu718, Ser719, Val726, Gly796, Cys797, Leu844, and Thr854. Mangiferin established two hydrogen bonds with the backbone atoms of Leu788 and Arg841 (Fig. 2D). Silybin formed one hydrogen bond with the side chain atom of Ser719, and three hydrogen bonds with the backbone atoms of Leu718, Cys797, and Phe795 (Fig. 2E). By contrast, gefitinib did not form hydrogen bonds (Fig. 2F).

### Molecular dynamics simulations of HER2–ligand interactions

**Fig. 3:**
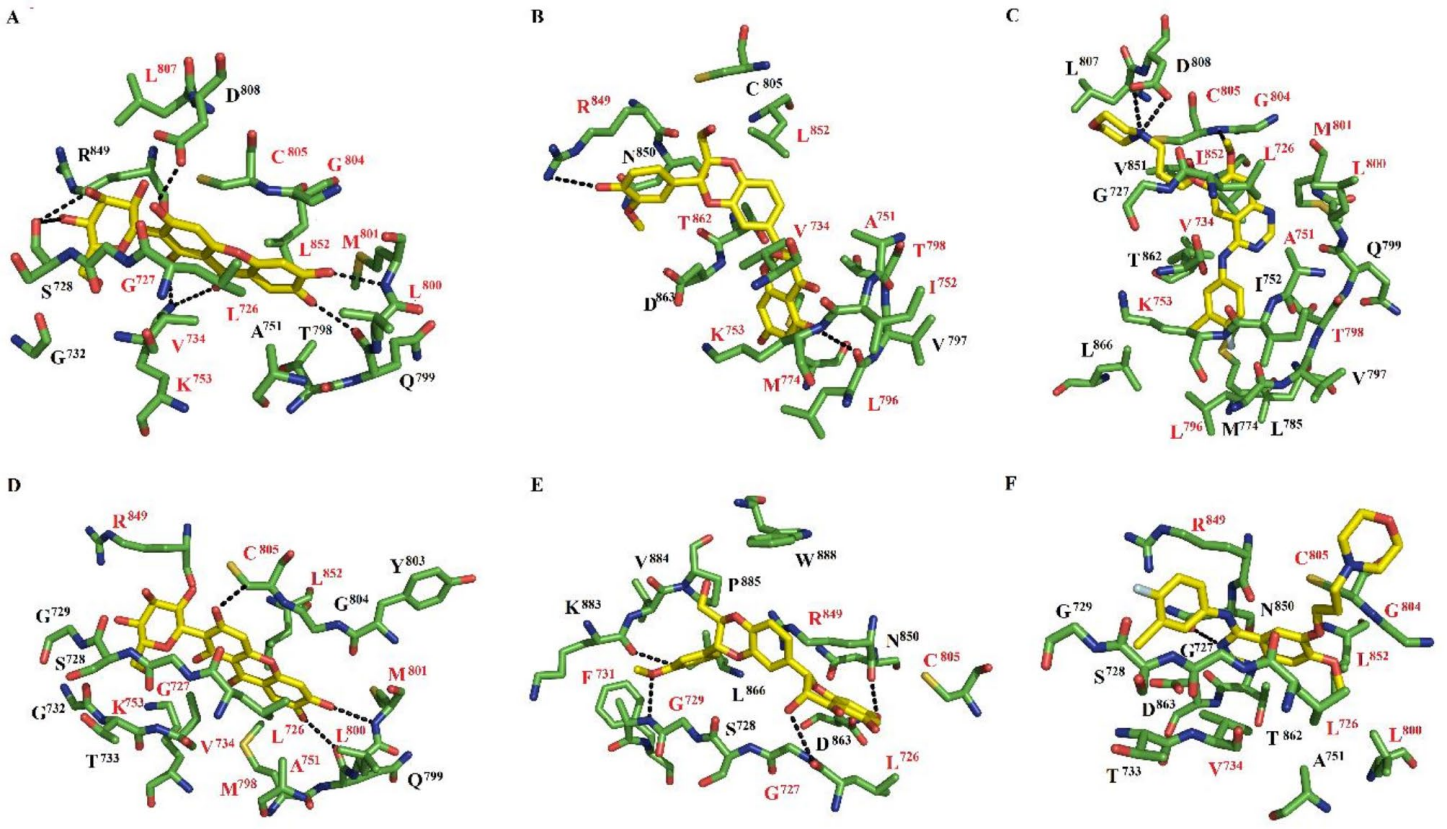
The receptor–ligand conformations for HER2–mangiferin (**A**), HER2–silybin (**B**), HER2–gefitinib (**C**), HER2_T798M_–mangiferin (**D**) HER2_T798M_–silybin (**E**), and HER2_T798M_–gefitinib (**F**).

The interactions between HER2 and mangiferin, silybin, or gefitinib were stabilized by 14–22 residues (Table S1). Among these residues, Va734, Ala751, Lys753, Thr798, Cys805, and Leu852 were consistently present in most interactions. Mangiferin established three hydrogen bonds with the side chain atoms of Lys753 and Asp808, and three hydrogen bonds with the backbone atoms of Ser728, Gln799, and Met801(Fig. 3A). Silybin formed a single hydrogen bond with the side chain atom of Arg849, and one hydrogen bond with the backbone atoms of Leu796 (Fig. 3B). Gefitinib created two hydrogen bonds with the side chain atom of Arg808, and one hydrogen bond with the backbone atoms of Cys805 (Fig. 3C).

The interactions between HER2_T798M_ and mangiferin, silybin, or gefitinib involved 15–18 residues (Table S1). Among these amino acid residues, Leu726, Gly727, Ser728, Gly729, Cy805, and Arg849 were consistently present in most complexes. Mangiferin established two hydrogen bonds with the backbone atoms of Gln799 and Met801 (Fig. 3D). Silybin formed four hydrogen bonds with the backbone atoms of Ala730 (Fig. 3E). Gefitinib formed one hydrogen bond with the side chain atom of Asn850 (Fig. 3F).

### ΔGbind calculations using the MMGBSA method

We calculated ΔG_bind_ by using the MMGBSA method. Table 1 shows that all systems exhibited a thermodynamically favorable ΔG_bind_. The nonpolar contributions from the van der Waals energy (ΔE_vdw_) and the nonpolar desolvation energy (ΔG_npol, sol_) guided the binding of the complexes. Comparative analysis of the complexes formed between mangiferin, silybin, or gefitinib and native or mutated EGFR, and the complexes formed between mangiferin, silybin, or gefitinib and native or mutated HER2 revealed that, although mangiferin and silybin showed favorable affinities for EGFR and HER2 in their native and mutated states, gefitinib showed higher affinity. Moreover, mangiferin and silybin showed similar affinity for most of the receptors, except silybin showed greater affinity for EGFR_T790−L858R_ than mangiferin did.

### Per-residue free energy decomposition

#### Per-residue free energy decomposition for native and wild-type EGFR–ligand complexes

We found that the complexes formed between EGFR and mangiferin, silybin, or gefitinib were stabilized by 18–19 amino acid resides (Table S2). When considering only those residues that exhibited a per-residue contribution of ≥ − 0.9 kcal/mol, we identified 11 residues contributing the most to ΔG_bind_ for EGFR–mangiferin: Leu718, Val726, Ala743, Lys745, Thr790, Leu792, Cys797, Asp800, Leu844, Thr854, and Asp855. Of these, the majority formed hydrophobic contacts, except for Asp800, Thr854, and Asp855, all of which formed hydrogen bonds with mangiferin (Fig. 1A). The residues that contributed the most to ΔG_bind_ for EGFR–silybin included Leu718, Val726, Gly796, Cys797, Leu844, Phe997 and Leu1001. These residues, except for Cys797, established hydrophobic contacts with silybin (Fig. 1B). Finally, Leu718, Val726, Ala743, Thr790, Leu792, Met793, Cys797, and Leu844 contributed the most to ΔG_bind_ for EGFR–gefitinib (Fig. 1C).

**Table 1:** The ΔG_bind_ components for the wild-type and mutant EGFR–ligand complexes after 100-ns molecular dynamics simulations (kcal/mol).

Systems	ΔE_vdw_	ΔE_ele_	ΔG_ele, sol_	ΔG_npol, sol_	ΔG_bind_
EGFR_− Mangiferin_	-43.36 (4.1)	-62.96 (8.5)	79.02 (6.1)	-6.36 (0.18)	-33.67 (3.6)
EGFR_− Silybin_	-40.85 (4.5)	-35.37 (8.0)	53.07 (7.0)	-5.84 (0.40)	-28.99 (4.1)
EGFR_− Gefitinib_	-50.81 (3.2)	-135.23 (16.1)	150.10 (15.0)	-6.37 (0.33)	-42.33 (4.0)
EGFR_L858R−Mangiferin_	-45.53 (3.5)	-41.77 (8.0)	62.54 (8.5)	-6.30 (0.22)	-31.08 (3.5)
EGFR_L858R−Silybin_	-53.07 (3.2)	-34.80 (7.5)	60.52 (6.5)	-6.95 (0.20)	-34.30 (3.4)
EGFR_L858R−Gefitinib_	-47.75 (3.1)	-121.99 (14.0)	135.95 (13.0)	-6.13 (0.32)	-39.94 (3.5)
EGFR_T790M, L858R−Mangiferin_	-36.51 (3.3)	-24.85 (9.5)	46.72 (8.7)	-5.06 (0.35)	-19.71 (3.2)
EGFR_T790M, L858R−Silybin_	-44.40 (3.1)	-36.06 (8.7)	46.27 (7.0)	-6.10 (0.25)	-32.71 (3.5)
EGFR_T790M, L858R−Gefitinib_	-54.35 (3.0)	-116.14 (14.0)	130.75 (13.5)	-6.87 (0.30)	-46.62 (3.8)
EGFR_G719S−Mangiferin_	-40.04 (3.9)	-50.43 (12.0)	68.57 (10.0)	-5.70 (0.17)	-27.62 (4.0)
EGFR_G719S−Silybin_	-48.12 (3.5)	-35.51 (11.0)	58.54 (8.0)	-6.29 (0.30)	-30.79 (3.1)
EGFR_G719S−Gefitinib_	-49.72 (3.0)	-145.91 (14.0)	158.74 (13.0)	-6.50 (0.35)	-43.40 (3.5)
HER2_− Mangiferin_	-42.55 (3.5)	-43.52 (9.0)	64.34 (8.0)	-5.75 (0.50)	-27.49 (4.0)
HER2_− Silybin_	-51.85 (3.0)	-31.70 (6.5)	58.33 (5.0)	-6.77 (0.30)	-32.00 (3.0)
HER2_− Gefitinib_	-68.72 (4.3)	-20.93 (14.0)	35.97 (14.0)	-8.7 (0.30)	-50.05 (4.1)
HER2_− T798M−Mangiferin_	-46.85 (3.3)	-43.64 (11.0)	65.53 (10.4)	-6.32 (0.35)	-31.29 (3.6)
HER2_− T798M−Silybin_	-45.47 (4.3)	-38.84 (10.5)	59.31 (8.5)	-6.07 (0.45)	-31.09 (4.0)
HER2_− T798M−Gefitinib_	-53.62 (4.5)	-19.28 (10.0)	42.45 (11)	-7.03 (0.60)	-37.49 (4.5)

All the energies were averaged over 500 snapshots and are in kcal/mol (± standard error of the mean).

The complexes formed between EGFR_L858R_ and mangiferin, silybin, or gefitinib were stabilized by 16–19 residues (Table S3). We identified 12 residues that contributed the most to ΔG_bind_ for EGFR_L858R_–mangiferin: Leu718, Val726, Ala743, Ile744, Lys745, Glu762, Thr790, Gly796, Cys797, Arg841, Leu844, and Thr854. Most of them formed hydrophobic contacts, except for Glu762, Arg841, and Cys797 that established hydrogen bonds with mangiferin (Fig. 1D). Leu718, Phe723, Val726, Lys745, Phe795, Cys797, Leu844, and Leu1001 contributed the most to ΔG_bind_ for EGFR_L858R_–silybin. Most of these residues formed hydrophobic contacts, except for Phe723, Leu718, Cys797, and Phe795, which established hydrogen bonds with silybin (Fig. 1E). Finally, Leu718, Ala743, Thr790, Leu792, Met793, Gly796, and Leu844 contributed the most to ΔG_bind_ for EGFR_L858R_–gefitinib. Most of them formed hydrophobic contacts, except for Gly796 that formed hydrogen bonds with gefitinib (Fig. 1F).

The complexes formed between EGFR_T790M−L858R_ and mangiferin, silybin, or gefitinib involved 16–18 residues (Table S4). Of these, Leu718, Gly719, Val726, Leu792, Cys797, Arg841, and Leu844 contributed the most to ΔG_bind_ for EGFR_T790M−L858R–_mangiferin. Most of them formed hydrophobic contacts, except Arg841 that established hydrogen bonds with mangiferin (Fig. 2A). Leu718, Gly719, Ser720, Val726, Lys745, Leu792, Met793, Leu844, and Thr854 contributed the most to ΔG_bind_ for EGFR_T790M−L858R_–silybin. Of these, most of them formed hydrophobic contacts, except Ser720, Lys745, L718, and M793, which established hydrogen bonds (Fig. 2B). Leu718, Val726, Ala743, Lys745, Leu788, Met790, Leu792, Met793, Gly796, Cys797, and Leu844 contributed the most to ΔG_bind_ for EGFR_T790M−L858R_–gefitinib (Table S4). All of them formed hydrophobic contacts (Fig. 2C).

The complexes formed between EGFR_G719S_ and mangiferin, silybin, or gefitinib involved 12–18 residues that contributed to ΔG_bind_ (Table S5). Of these, Leu718, Val726, Lys745, Gly796, Cys797, Arg841, and Leu844 contributed the most to ΔG_bind_ for EGFR_G719S_–mangiferin. They formed hydrophobic contacts, except for R841, which established hydrogen bonds (Fig. 2D). Leu718, Val726, Gly796, Cys797, Arg841, and Leu844 contributed the most to ΔG_bind_ for EGFR_G719S_–silybin. Most of them formed hydrophobic contacts, except L718 and Cys797 that established hydrogen bonds (Fig. 2E). Finally, Leu718, Val726, Ala743, Met790, Leu792, Met793, Gly796, and Leu844 contributed the most to ΔG_bind_ for EGFR_G719S_–gefitinib. All of them formed hydrophobic contacts (Fig. 2F).

#### Per-residue free energy decomposition for native and wild-type HER2–ligand complexes

The HER2–mangiferin complex involved 17 amino acid residues (Table S6). However, considering a per-residue contribution − 0.9 kcal/mol, Leu726, Gly727, Val734, Lys753, Leu800, Met801, Gly804, Cys805, Leu807, and Leu852 contributed the most to ΔG_bind_ for this complex. Most of them formed hydrophobic contacts, except Lys753, which formed hydrogen bonds (Fig. 3A). Val734, Ala751, Ile752, Lys753, Met774, Leu796, Thr798, Arg849, Leu852, and Thr862 contributed the most to ΔG_bind_ for HER2–silybin. Most of them formed hydrophobic contacts, except for Leu796 and Arg849, which established hydrogen bonds (Fig. 3B). Finally, Leu726, Val734, Ala751, Lys753, Leu796, Thr798, Leu800, Met801, Gly804, Cys805, and Leu852 contributed the most to ΔG_bind_ for HER2–gefitinib. Almost all of them formed hydrophobic contacts (Fig. 3C), except for Cys805, which formed hydrogen bonds.

The following residues contributed the most to ΔG_bind_ for HER2_T798M_–mangiferin: Leu726, Gly727, Val734, Ala751, Lys753, Thr798, Leu800, Met801, Cys805, Arg849, and Leu852 (Table S7, Supplementary Material). Most of them formed hydrophobic contacts, except for Met801 that formed hydrogen bonds with mangiferin (depicted in Fig. 3D). For HER2_T798M_–silybin, Leu726, Gly727, Gly729, Phe731, Cys805, and Arg849 contributed the most to ΔG_bind_; they formed hydrophobic contacts (Fig. 3E). Finally, for HER2_T798M_–gefitinib, Leu726, Val734, Leu800, Cys805, Arg849, and Leu852 contributed the most to ΔG_bind_; all of them formed hydrophobic contacts (Fig. 3F).

### Physicochemical analysis based on DFT

Molecular electronic properties and interactions are inherently linked to molecular structure; hence, structural optimization is essential to accurately predict these electronic characteristics and interactions^36,37^. DFT has proven to be highly effective in refining molecular structures and reliably forecasting electronic properties, which are crucial indicators for drug activity^38,39^. Consequently, we optimized the structures of silybin, mangiferin, and gefitinib by using the Gaussian 09 software^31^.

**Fig. 4:**
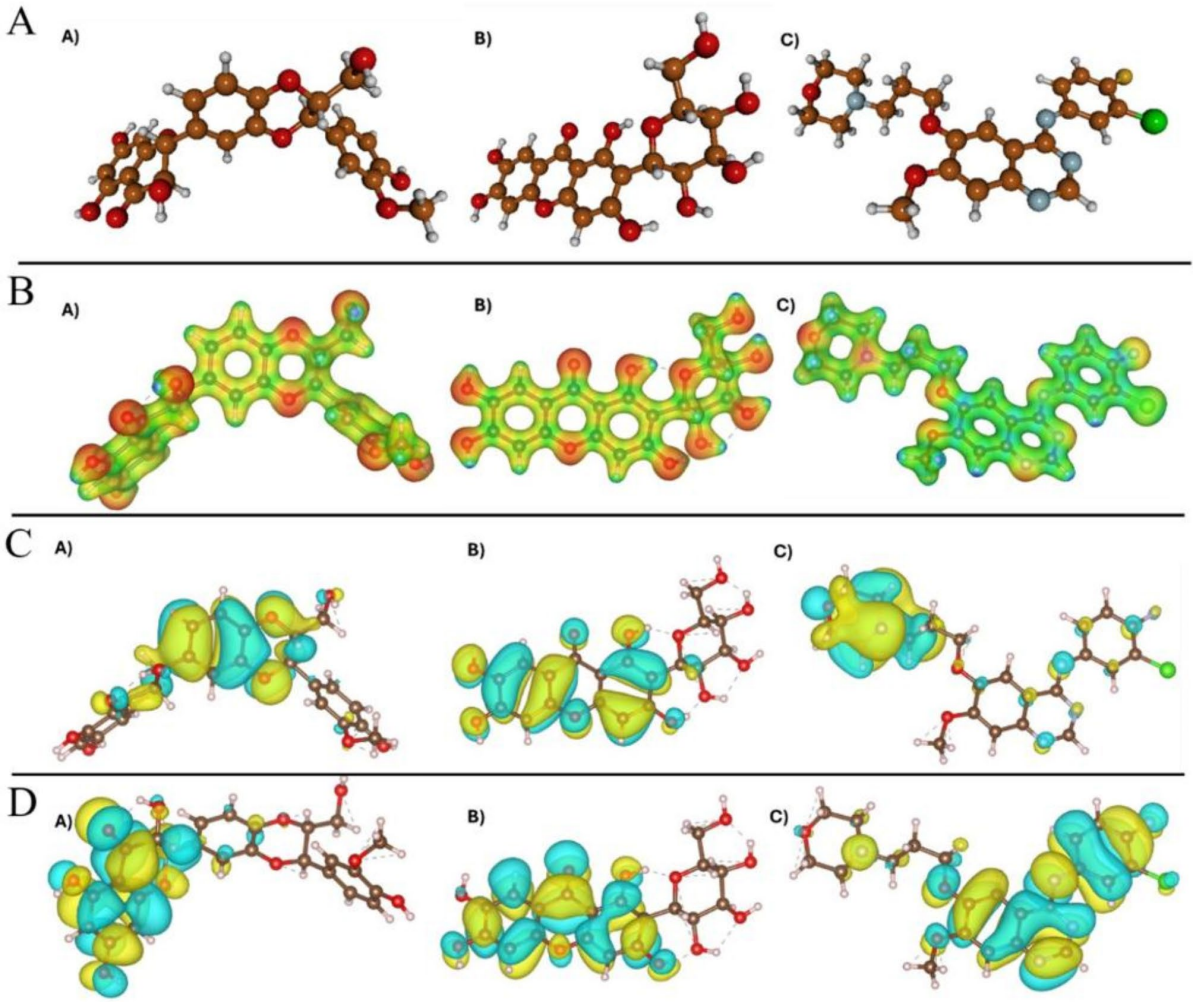
Graphical representation of the energy optimized structures (**A**), MEP maps (**B**), HOMO (**C**), and LUMO (**D**) of silybin (**A**), mangiferin (**B**), and gefitinib (**C**).

This analysis focused on generating MEP maps and FMOs, including the HOMO and the LUMO. A MEP map shows the spatial distribution of molecular charges, with red representing negative charges, green neutral charges, and blue positive charges. HOMO and LUMO energy levels define a molecule’s electron donation and acceptance capabilities, respectively; HOMO indicates regions that can donate electrons, while LUMO reflects electron-accepting potential^40^. Importantly, a higher LUMO energy has been associated with enhanced target binding due to its electron-accepting abilities^38,40^.

Figure 4 illustrates the energy optimized structures (Fig. 4A), MEP, and HOMO and LUMO. For MEP analysis, molecular regions prone to electrophilic attacks, indicated by blue surfaces, are mainly located around hydrogen atoms. Conversely, red surfaces highlight areas more susceptible to nucleophilic attacks, typically around oxygen atoms. Specifically, mangiferin and silybin, similarly to antioxidant molecules, exhibit more -OH groups, which may enhance the hydrogen bond formation potential (Fig. 4B), in contrast to gefitinib.

Silybin is a flavonolignan compound characterized by a complex structure that includes a 2,3-dihydro-1,4-benzodioxin core with a hydroxymethyl group attached at the 2-position. This core is linked to two distinct aromatic rings: one bearing a 4-hydroxy-3-methoxyphenyl group and the other bearing a 4-methoxyphenyl group (Fig. 4A-a). Silybin exhibits HOMO localized on the 2,3-dihydro-1,4-benzodioxin core, in contrast to LUMO located on the 4-hydroxy-3-methoxyphenyl group (Fig. 4C-a and D-a).

Mangiferin is a C-glycosyl compound consisting of 1,3,6,7-tetrahydroxyxanthen-9-one having a beta-D-glucosyl residue at the 6-position (Fig. 4A-b). Mangiferin exhibits a homogeneous orbital distribution, with HOMO and LUMO appearing in the xanthan motif, suggesting that this motif is more susceptible to nucleophilic and electrophilic attack than the beta-D-glucosyl residue (Fig. 4C-b and D-b).

Gefitinib is a quinazoline that is substituted by a (3-chloro-4-fluorophenyl)nitril group, 3-(morpholin-4-yl)propoxy group and a methoxy group at positions 4, 6, and 7 (Fig. 4A-c). The HOMO and LUMO are polarized on the gefitinib structure: The HOMO is associated with the –(morpholin-4-yl)propoxy group, while the LUMO is located on the quinazoline core and the (3-chloro-4-fluorophenyl) nitril group.

The HOMO and LUMO energy levels are fundamental for calculating electronic descriptors that offer insights into molecular reactivity. We used the TAFF software^41^ to compute these values (Table 2). We found higher values for gefitinib compared with mangiferin and silybin. This could be correlated with higher binding energies calculated after molecular simulation and could be associated with binding energies in absence of hydrogen bond formation.

The highest HOMO energy was for gefitinib (–194.10 kcal/mol), followed by silybin (–138.70 kcal/mol) and mangiferin (–133.14 kcal/mol), indicating that gefitinib has the greatest capacity to donate electrons. Gefitinib also had the highest LUMO energy (–184.45 kcal/mol), followed by silybin (–38.85 kcal/mol) and mangiferin (–34.81 kcal/mol) suggesting that gefitinib has the greatest electron-accepting capability, a key factor in molecular interaction efficiency. The HOMO-LUMO gap energy (E_LUMO−HOMO_) is associated with kinetic stability and chemical reactivity; a small gap is associated with low kinetic stability and high chemical reactivity^42,43^. Gefitinib had the lowest gap energy (9.25 kcal/mol), in contrast to silybin (–38.85 kcal/mol) and mangiferin (–34.81 kcal/mol).

We also analyzed electron affinity, ionization energy, electronegativity, electrophilicity indices, global hardness and softness, and chemical potential. As mentioned above, gap energy indicates a molecule’s stability. Chemical potential measures electron transfer within the system^44,45^. Net electrophilicity incorporates both gap energy and chemical potential and is an effective predictor of reactivity, especially in covalent inhibitors. A low gap energy and high chemical potential yield a high net electrophilicity, signaling enhanced reactivity. Of the three considered compounds, gefitinib had the highest net electrophilicity (7762.88 kcal/mol) due to its low gap energy (9.25 kcal/mol) and high chemical potential (–189.47 kcal/mol).

**Table 2:** The reactivity indexes determined for Silybin, mangiferin, and gefitinib.

Reactivity Index	Gefitinib*	Mangiferin*	Silybin*
HOMO energy	-194.10	-133.14	-138.70
LUMO energy	-184.85	-34.81	-38.85
GAP	9.25	98.33	99.85
Ionization potential	194.10	133.14	138.70
Electroaffinity	184.85	34.81	38.85
Chemical potential	-189.47	-83.98	-88.77
Global Hardness	4.63	49.17	49.93
Global Softness	42566.48	4004.33	3943.37
Electronegativity	189.47	83.98	88.77
Electrophilicity Index (w)	3880.86	71.72	78.92
w + Electron Acceptor	3786.70	35.87	40.78
w- Electron Donator	3976.17	119.85	129.55
Electrophilicity Net	7762.88	155.72	170.33

* indicates Kcal/mol.

### Silybin and mangiferin inhibit breast cancer cell proliferation

**Fig. 5:**
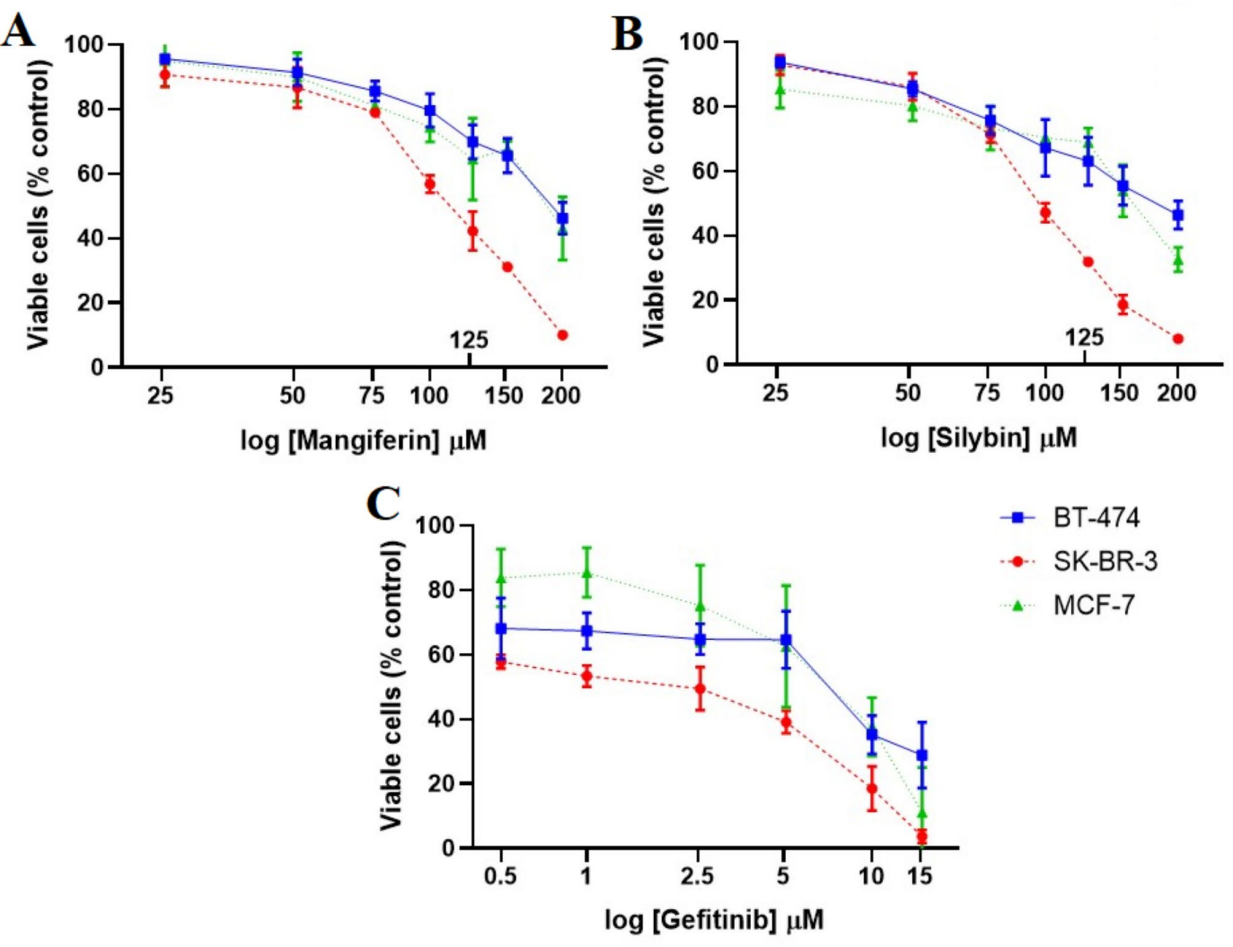
The percentage of viable BT-474, and SK-BR-3 cells (HER2-positive breast cancer cell lines) after treatment with (**A**) mangiferin, (**B**) silybin, and (**C**) gefitinib. Each data point is the mean ± standard deviation of three independent experiments.

We evaluated the in vitro effect of silybin and mangiferin in two HER2-positive breast cancer cell lines, BT-474 and SK-BR-3. We treated the cells in a dose-dependent manner from 0 to 200 µM silybin or mangiferin or 0–15 µM reference drug gefitinib to determine the IC_50_. We treated the cells with 0–200 µM silybin or mangiferin or 0–15 µM gefitinib to determine the IC_50_. The IC_50_ of silybin and mangiferin were substantially different between SK-BR-3 and BT-474 cells: For BT-474, they were 178.4 and 195.2 µM, respectively, approximately twice as high for SK-BR-3 cells (96.62 and 110.8 µM, respectively; Fig. 5; Table 3). By contrast, SK-BR-3 and BT-474 cells were much more sensitive to gefitinib, with an IC_50_ of 1.31 and 5.01 µM, respectively (Fig. 5; Table 3).

**Table 3:** Cytotoxic activity (IC_50_ in M*) in three breast cancer cell lines after incubation for 48 h.

	BT-474	SK-BR-3
IC_50_ Mangiferin [µM]	195.2	110.8
IC_50_ Silybin [µM]	178.4	96.62
IC_50_ Gefitinib [µM]	5.01	1.31

* The IC_50_ of each compound was determined based on the curve of cell viability percentage versus the logarithmic of concentration.

## Discussion

It is crucial to identify natural and dual HER2 and EGFR inhibitors in the fight against HER2-positive and EGFR-driven cancers. In this sense, mangiferin and silybin are two natural compounds that have demonstrated encouraging results as anticancer drugs^14–17^. However, prior to our study, there had been no structural information about the binding of these two compounds to wild-type HER2 or EGFR, or HER2 or EGFR with mutations in the ATP binding site that confer primary resistance to tyrosine kinase inhibitors. We utilized a combination of molecular docking, molecular dynamics simulations combined with the MMGBSA method, DFT analysis, and cell culture assays to evaluate the inhibitory properties of silybin and mangiferin on EGFR or HER2. Docking analysis demonstrated the ability of mangiferin, silybin, and the reference compound, gefitinib, to bind to the ATP binding site, whereas triplicate 100-ns-long molecular dynamics simulations for each system showed preservation of the predicted^18,46–50^. For all complexes, mangiferin and silybin formed more hydrogen bonds than gefitinib (Figs. 1, 2 and 3), consistent with the HOMO and LUMO analysis (Fig. 4).

Structural analysis of the most populated complexes obtained through molecular dynamics simulations of mangiferin, silybin, or gefitinib binding to EGFR showed that silybin, mangiferin, and gefitinib form a complex with EGFR via interactions with Thr790 and Met793. Interaction with Thr790 is characteristic of different EGFR tyrosine kinase inhibitors, and mutation of this residue impacts the affinity of the inhibitor^9,51–53^. Interaction with Met793 is key for ligand stabilization and has previously been observed for other EGFR inhibitors^47,50^. For the complexes between the three compounds with EGFR_L858R_, it was observed that silybin, mangiferin, and gefitinib interact with Gly719 and Thr790^9,50–53^, indicating that L858R promotes interactions between the evaluated compounds and Gly719 and Thr790. These two residues are susceptible to mutation and have been linked to medication resistance by destabilizing the inactive conformation^9^.

For the complexes with EGFR_T790M−L858R_, it was observed that silybin, mangiferin, and gefitinib interact with Gly719, Met790, and Met793. The participation of these three residues in the stabilization of the three complexes indicates that their mutation directly impacts the map of interactions.

For the complexes with EGFR_G719S_, it was found that mangiferin, similarly to gefitinib, interacted with Thr790 but not Met793, while silybin did not interact with either of those residues. All three compounds interacted with G719S, indicating that this mutation impacts the interactions of the compounds with G719S, Thr790, and Met793.

For the complexes with HER2, all three compounds interact with Thr798, a characteristic interaction for several HER2 tyrosine kinase inhibitors; mutation of this residue impacts the affinity for the inhibitor. Another important residue in the stabilization of tyrosine kinase inhibitors is Met801, although this residue was only involved in stabilization of the HER2–mangiferin complex. For the complexes with HER2_T798M_, eleven amino acids (Leu726, Gly727, Ser728, Gly729, Thr733, Val734, Ala751, Leu800, Cys805, Arg849, and Leu852) participated in stabilization of the mangiferin–HER2_T798M_ and gefitinib-HER2_T798M_ complexes, while there were only six shared amino acids (Leu726, Gly727, Ser728, Gly729, Cys805, and Arg849) for silybin and gefitinib, indicating a greater inhibitory potential of HER2_T798M_ for mangiferin than for silybin. However, mangiferin interacted with Thr798, a characteristic interaction for different HER2 tyrosine kinase inhibitors, so the affinity of mangiferin for HER2_T798M_ would be more affected than the affinity of silybin for HER2_T798M_.

In overall, structural analysis of the most populated complexes obtained through molecular dynamics simulations of mangiferin, silybin, or gefitinib binding to EGFR, EGFR_L858R_, EGFR_T790M−L858R_, or EGFR_G719S_ revealed a decrease in the number of residues stabilizing the EGFR_L858R_–ligand, EGFR_T790M−L858R_–ligand, or EGFR_G719S_–ligand complexes with respect to the EGFR–ligand complexes, especially for the EGFR_G719S_–ligand complexes. Considering only contacts with Gly/Ser719, Thr/Met790, and Met793, the EGFR–ligand complexes maintained interactions with Thr790 and Met793, while the EGFR_T790M−L858R_–ligand complexes preserved interactions with Gly719, Met790, and Met793. The EGFR_L858R_–ligand complexes preserved interactions with Gly719 and Thr790, whereas the EGFR_G719S_–ligand complexes only preserved interactions with Gly/Ser719. This analysis suggests that the binding of silybin and mangiferin is less affected by the EGFR_L858R_ or EGFR_G719S_ mutation than the EGFR_T790M−L858R_ mutations.

The most populated complexes of mangiferin, silybin, or gefitinib with HER2 or HER2_T798M_ showed that these compounds interact with Thr798, but this interaction is lost when for silybin and gefitinib when this residue is mutated to methionine (i.e., in the HER2_T798M_–silybin and HER2_T798M_–gefitinib complexes). Hence, T798M has a greater impact on the affinity between mangiferin and HER2_T798M_ compared with the affinity between silybin and HER2_T798M_.

ΔG_bind_ calculated with the MMGBSA method was favorable for the formation of the complexes between mangiferin or silybin and EGFR or HER2 in its native and mutated form, but in all cases gefitinib showed higher affinity, consistent with the reactivity indexes (Table 2). Interestingly, even when EGFR or HER2 was mutated, mangiferin or silybin exhibited almost similar binding affinity as with the wild-type receptors, suggesting higher stability of these compounds with respect to gefitinib at the ATP binding site. Consistently, the reactivity indexes suggest that the higher reactivity of gefitinib could allow stronger binding but lesser stable interactions (Table 2).

Per-residue analysis considering the residues that contributed the most to the binding affinity between mangiferin, silybin, or gefitinib and EGFR, EGFR_L858R_, EGFR_T790M−L858R_ revealed that Leu718, Val726, Cys797, and Leu844, present in the stabilization between EGFR and ATP^53^, were present in most of the systems, suggesting an important role of these residues in ligand stabilization. Although the per-residue contribution analysis indicated the participation of Gly/Ser719, Thr/Met790, and Met793 for most of the complexes, Thr/Met790 only participated for the complexes formed between mangiferin or gefitinib and EGFR and EGFR_L858R_. Moreover, mangiferin shared a higher number of common residues contributing the most to the ΔG_bind_ with gefitinib compared with the common residues between silybin and gefitinib. Met793 was only involved in the complexes formed between silybin or gefitinib and EGFR_T790M−L858R_. For this system, silybin shared a higher number of common contacts with gefitinib than with mangiferin. Gly719 only participated in the complexes formed between mangiferin or silybin and EGFR_T790M−L858R_, whereas the complexes formed between mangiferin, silybin, or gefitinib and EGFR_G719S_ did not involve Gly/Ser719, Thr790, or Met793. Interestingly, Thr/Met790 and Met793 contributed to all complexes with gefitinib.

Met801 only participated in complexes formed between mangiferin or gefitinib and HER2. Mangiferin shared a higher number of common contacts with gefitinib (Leu726, Val734, Lys753, Leu800, Met801, Gly804, Cys805, and Leu852) than with silybin (Val734, Ala751, Lys753, Leu796, Thr798, and Leu852). For complexes formed between mangiferin, silybin, or gefitinib and HER2_T798M_, Met801 and Thr798 only participated in the HER2_T798M_–mangiferin complex. In addition, mangiferin shared more common contacts with gefitinib (Leu726, Val734, Leu800, Cys805, Arg849, and Leu852) than with silybin (Leu726, Cys805, and Arg849).

The electronic properties and reactivity indices of silybin, mangiferin, and gefitinib evaluated using Density Functional Theory (DFT) calculations providing valuable insights into their binding behavior and stability in interactions with EGFR and HER2. The MEP analysis explains why mangiferin and silybin form hydrogen bonds with EGFR, EGFRL858R, EGFR_T790M−L858R_, EGFR_G719S_, HER2, and HER2_T798M_, whereas gefitinib does not (Figs. 1, 2 and 3). The molecular orbitals of silybin suggest that its core is more prone to donating electrons, while the 4-hydroxy-3-methoxyphenyl group is more likely to accept electrons when complexed with EGFR, EGFR_L858R_, EGFR_T790M−L858R_, EGFR_G719S_, HER2, and HER2_T798M_. This electronic behavior aligns with the hydrogen bonds observed in these complexes. Similarly, the molecular orbitals of mangiferin support the formation of hydrogen bonds between its xanthan motif and EGFR, EGFR_L858R_, EGFR_T790M−L858R_, EGFR_G719S_, HER2, and HER2_T798M_, while the beta-D-glucosyl residue interacts through hydrogen bonding only with EGFR_T790M−L858R_ and HER2 (Figs. 1, 2 and 3).

HOMO and LUMO energy levels play a crucial role in determining a molecule’s ability to donate and accept electrons, which directly impacts its binding affinity. Among the studied compounds, gefitinib exhibited the highest HOMO and LUMO energy levels, suggesting a greater capacity for electron donation and acceptance compared to silybin and mangiferin. The smaller HOMO-LUMO gap observed for gefitinib (9.25 kcal/mol) indicates higher reactivity, which corresponds to stronger but less stable interactions with EGFR and HER2. This observation is consistent with molecular dynamics simulations, which demonstrated higher binding energies for gefitinib in both wild-type and mutant forms of EGFR and HER2 (Table 2). The increased reactivity of gefitinib is associated with more dynamic yet less stable interactions at the ATP binding site.

In contrast, silybin and mangiferin exhibited lower reactivity, as indicated by their larger HOMO-LUMO gaps and more stable binding profiles, particularly with mutated forms of EGFR (EGFR_L858R_ and EGFR_G719S_) and HER2 (HER2_T798M_). The stability of the complexes formed by these compounds, despite mutations at critical binding sites, suggests their potential as more stable inhibitors capable of overcoming resistance-associated mutations. The reactivity indices indicate that gefitinib’s higher reactivity enables stronger but transient interactions with EGFR, EGFR_L858R_, EGFR_T790M−L858R_, EGFR_G719S_, HER2, and HER2_T798M_. Conversely, the lower reactivity indices of mangiferin and silybin suggest greater kinetic stability, leading to more durable interactions with these proteins.

Interestingly, while gefitinib demonstrated higher reactivity and binding affinity, mangiferin and silybin formed a greater number of hydrogen bonds with EGFR and HER2, a finding consistent with the HOMO and LUMO analyses. These results align with docking and molecular dynamics simulations, which confirmed that mangiferin and silybin establish more stable hydrogen bonds with key residues of EGFR and HER2, particularly in the presence of mutations such as EGFR_L858R_ and EGFR_G719S_.

Structural flexibility plays a crucial role in ligand binding and stabilization, as revealed by the RMSF analysis. Our simulations showed that wild-type EGFR and HER2 exhibited higher mobility in key loop regions, particularly in the β-sheet 7 – α-helix 4 and α-helix 5 – α-helix 6 loops, which are associated with ATP binding and kinase activation. However, mutant EGFR (L858R, G719S, and T790M-L858R) and mutant HER2 (T798M) showed reduced fluctuations in these regions, suggesting that these mutations stabilize the local conformation and alter ligand accommodation at the ATP-binding site. Notably, the EGFR_T790M−L858R_-ligand complex displayed distinct mobility patterns in the β-sheet 3 – α-helix 1 loop (747–752), which may impact ligand accessibility and binding dynamics. These findings correlate with our binding free energy (ΔG_bind_) calculations, where mangiferin and silybin maintained stable interactions across both wild-type and mutant receptors. Despite the rigidification observed in HER2_T798M_, mangiferin still formed key stabilizing interactions, while silybin showed a reduction in affinity. This suggests that structural flexibility constraints induced by mutations may differentially affect ligand stabilization, which should be considered in the optimization of new inhibitors targeting EGFR and HER2. Furthermore, the higher number of hydrogen bonds formed by mangiferin and silybin compared to gefitinib (as revealed by molecular docking and MD simulations) indicates that these natural compounds may compensate for reduced flexibility by engaging in stronger stabilizing interactions with key residues such as Thr790 in EGFR and Thr798 in HER2. The results highlight that mutations not only confer resistance to traditional kinase inhibitors but also reshape the dynamic landscape of the binding site, influencing ligand binding mechanisms.

According to the theoretical analysis and the results of the in vitro antiproliferative experiment, silybin and mangiferin showed a higher IC_50_ than gefitinib for both BT-474 and SK-BR-3 cells. Our results suggest that silybin and mangiferin have the potential to inhibit EGFR and HER2 activation, probably by preventing the formation of homo- and heterodimers of these receptors.

## Conclusions

MMGBSA calculations indicated favorable binding affinities for mangiferin and silybin with both wild-type and mutant forms of EGFR and HER2, suggesting greater stability at the ATP-binding site compared to gefitinib, in agreement with DFT reactivity indexes. Per-residue decomposition analysis revealed that both natural compounds exhibit inhibitory properties similar to gefitinib, with mangiferin showing superior inhibition over silybin. RMSF analysis revealed that mutations in EGFR and HER2 reduce structural flexibility, particularly in regions near the ATP-binding site, potentially affecting ligand accommodation. However, mangiferin and silybin maintained stable interactions despite these conformational changes, reinforcing their potential as dual EGFR/HER2 inhibitors. Despite their antiproliferative effects on BT-474 and SK-BR-3 breast cancer cells, their activity was lower than gefitinib. This structure-based analysis provides valuable insights into the potential of natural compounds as inhibitors of EGFR/HER2 in breast cancer therapy.

## Electronic supplementary material

Below is the link to the electronic supplementary material.

Supplementary Material 1

## Data Availability

The datasets generated and analyzed during the current study are available from the corresponding authors upon request.
