# Supplementary material for: Insight into the inhibitory activity of mangiferin and Silybin against HER2 and EGFR using theoretical and experimental approaches
Source: Sci Rep. 2025 Mar 13;15:8658. doi: 10.1038/s41598-025-93612-2 (PMC11906813; doi:10.1038/s41598-025-93612-2)
Supplement: Supplementary file 1 — Supplementary Material 1 [file 41598_2025_93612_MOESM1_ESM.docx]

**SUPPLEMENTARY MATERIAL**

Insight into the inhibitory activity of Mangiferin and Silybin compounds on HER2 and EGFR using theoretical and experimental approaches.

Jesús Eduardo Alvarado-Lozano,^1^ Jorge Arturo Hernández-Valencia,^1^ Rodolfo Daniel Ávila-Avilés,^2,3^ Martiniano Bello^1*^

^1^Laboratorio de Diseño y Desarrollo de Nuevos Fármacos e Innovación Biotecnológica, Sección de Estudios de Posgrado e Investigación, Escuela Superior de Medicina, Instituto Politécnico Nacional, Plan de San Luis y Salvador Diaz Mirón s/n, Casco de Santo Tomás, Miguel Hidalgo, Ciudad de México 11340, México.

^2^Transdisciplinary Research for Drug Discovery, Sociedad Mexicana de Epigenética y Medicina Regenerativa A. C. (SMEYMER), México City, México

^3^Centro Conjunto de Investigación en Química Sustentable (CCIQS), UAEM-UNAM. Toluca, Estado de México, 50200 México


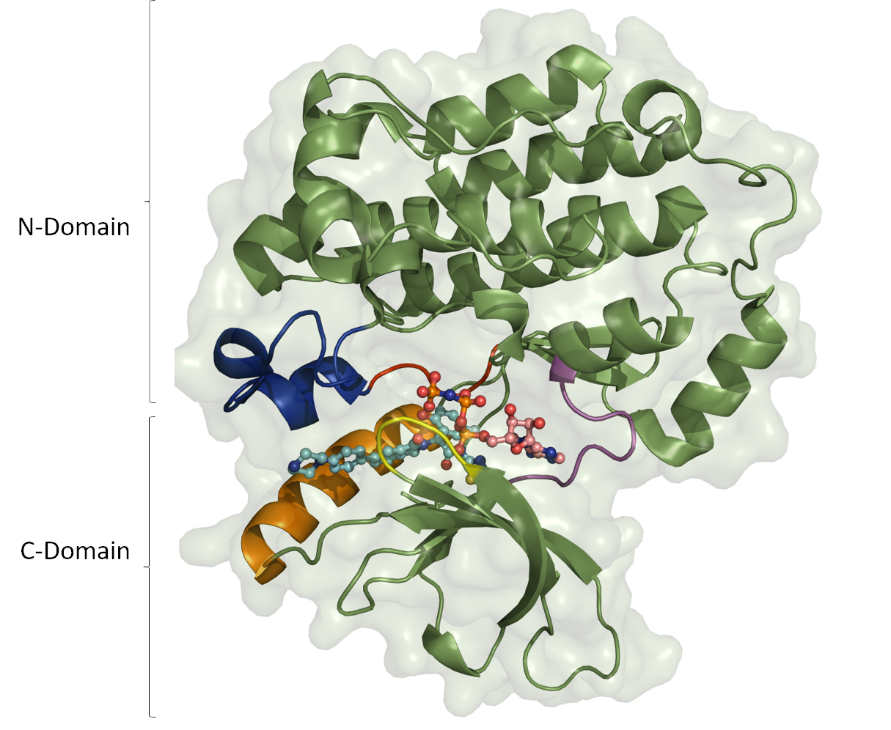


Fig. S1. Architecture of the catalytic domain of a protein kinase, namely epidermal growth factor receptor (EGFR; Protein Data Bank [PDB] entry 6DUK). The figure illustrates the N- and C-termini of EGFR forming a complex with adenosine triphosphate (ATP) and substrate at the catalytic binding domain. The activation loop is colored in blue, the P-loop in red, the αC-helix in orange, the hinge region in magenta, the ATP binding region in pink, and the substrate in cyan.


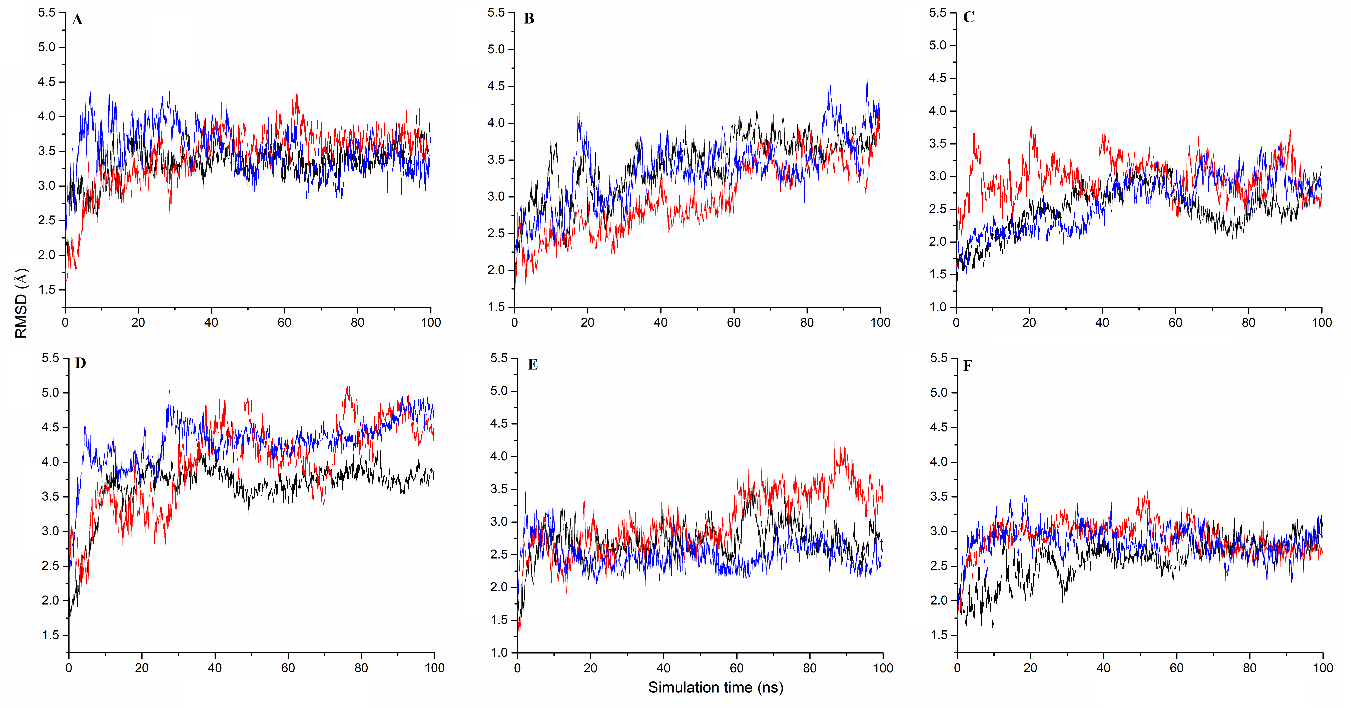
**Fig. S2.** RMSD analysis of receptor-ligand systems. A) EGFR-_Mangiferin_, EGFR-_Silybin_ or EGFR-_Gefitinib_, B) EGFR_L858R-Mangiferin_, EGFR_L858R-Silybin_ or EGFR_L858R-Gefitinib_, C) EGFR_T790M, L858R-Mangiferin_, EGFR_T790M, L858R-Silybin_ or EGFR_T790M, L858R-Gefitinib_, D) EGFR_G719S-Mangiferin_, EGFR_G719S-Silybin_ or EGFR_G719S-Grfitinib_, E) HER2-_Mangiferin_, HER2-_Silybin_ or HER2-_Gefitinib_, and F) HER2_T798M_-_Mangiferin_, HER2 _T798M_-_Silybin_ or HER2 _T798M_-_Gefitinib_. Complexes of EGFR or HER2 with Mangiferin, Silybin and Gefitinib are represented as black, red and blue lines.


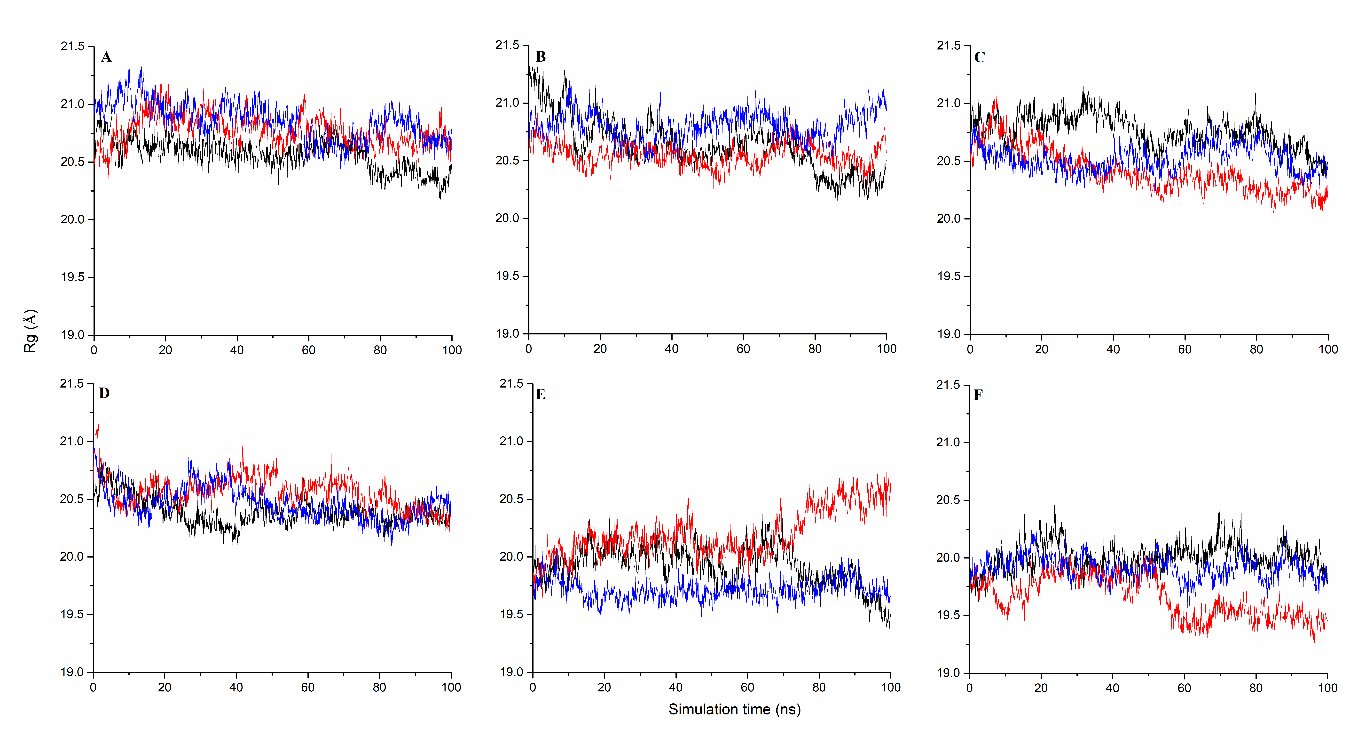
**Fig. S3.** Rg analysis of receptor-ligand systems. A) EGFR-_Mangiferin_, EGFR-_Silybin_ or EGFR-_Gefitinib_, B) EGFR_L858R-Mangiferin_, EGFR_L858R-Silybin_ or EGFR_L858R-Gefitinib_, C) EGFR_T790M, L858R-Mangiferin_, EGFR_T790M, L858R-Silybin_ or EGFR_T790M, L858R-Gefitinib_, D) EGFR_G719S-Mangiferin_, EGFR_G719S-Silybin_ or EGFR_G719S-Gefitinib_, E) HER2-_Mangiferin_, HER2-_Silybin_ or HER2-_Gefitinib_, and F) HER2_T798M_-_Mangiferin_, HER2 _T798M_-_Silybin_ or HER2 _T798M_-_Gefitinib_. Complexes of EGFR or HER2 with Mangiferin, Silybin and Gefitinib are represented as black, red and blue lines.


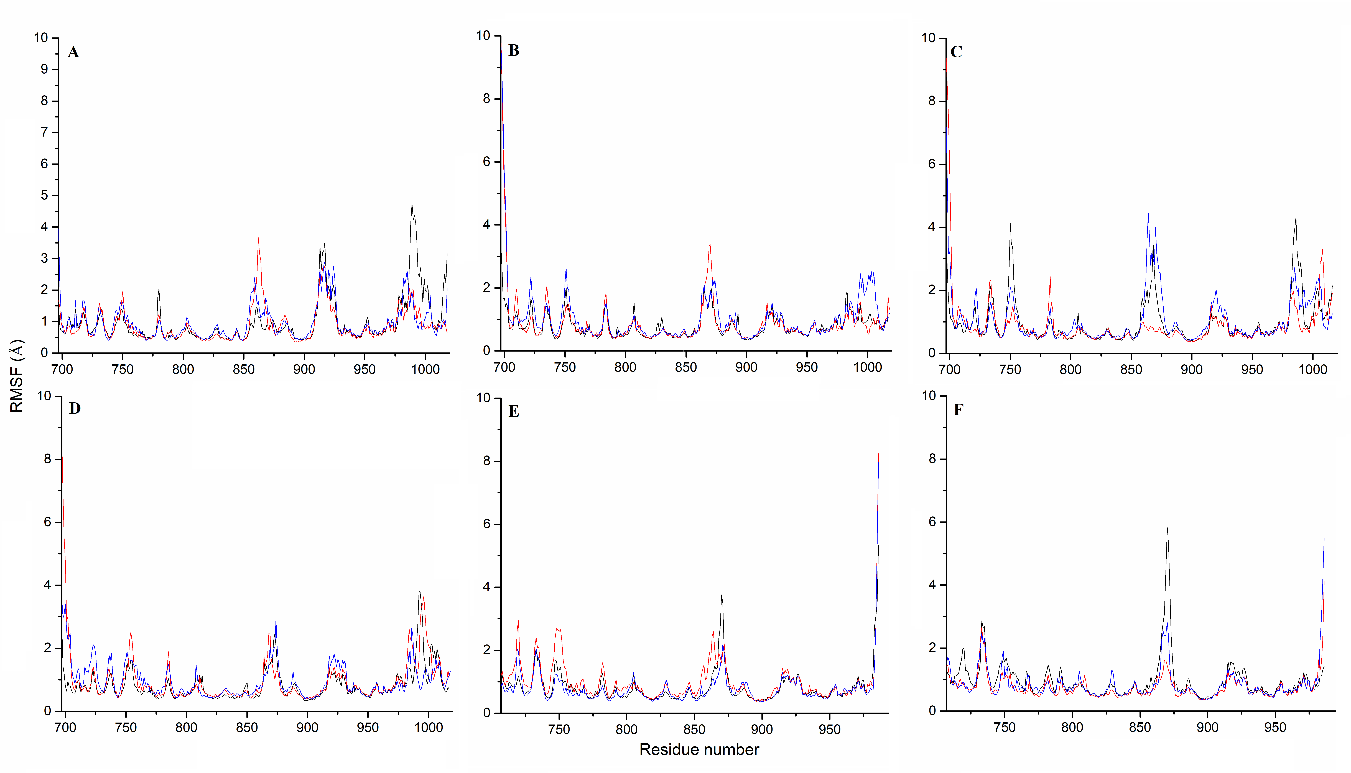


**Fig. S4.** RMSF analysis of receptor-ligand systems. A) EGFR-_Mangiferin_, EGFR-_Silybin_ or EGFR-_Gefitinib_, B) EGFR_L858R-Mangiferin_, EGFR_L858R-Silybin_ or EGFR_L858R-Gefitinib_, C) EGFR_T790M, L858R-Mangiferin_, EGFR_T790M, L858R-Silybin_ or EGFR_T790M, L858R-Gefitinib_, D) EGFR_G719S-Mangiferin_, EGFR_G719S-Silybin_ or EGFR_G719S-Gefitinib_, E) HER2-_Mangiferin_, HER2-_Silybin_ or HER2-_Gefitinib_, and F) HER2_T798M_-_Mangiferin_, HER2 _T798M_-_Silybin_ or HER2 _T798M_-_Gefitinib_. Complexes of EGFR or HER2 with Mangiferin, Silybin and Gefitinib are represented as black, red and blue lines.

**Table S1**. List of residues and type of interactions present in the complexes between Mangiferin, Silybin, or Gefitinib with wild-type and mutant EGFR or HER2.

| Sistema | Van der Waals |
| --- | --- |
| EGFR_-Mangiferin_ | Leu718, Val726, Ala743, Ile744, Lys745, Leu777, Leu788, Ile789, Thr790, Gln791, Leu792, Met793, Gly796, Cys797, Asp800, Leu844, Thr854, Asp855, Leu1001 |
| EGFR_-Silybin_ | Leu718, Gly719, Phe723, Val726, Ala743, Lys745, Thr790, Gln791, Leu792, Met793, Phe795, Gly796, Cys797, Asp800, Leu844, Thr854, Phe997, Leu1001 |
| EGFR_-Gefitinib_ | Leu718, Gly719, Val726, Ala743, Ile744, Lys745, Leu777, Leu788, Ile789, Thr790, Gln791, Leu792, Met793, Gly796, Cys797, Leu844, Thr854, Leu1001, |
| EGFR_L858R-Mangiferin_ | Leu718, Gly719, Val726, Ala743, Ile744, Lys745, Glu762, Met766, Leu788, Thr790, Gly796, Cys797, Arg841, Asn842, Leu844, Thr854, Asp855 |
| EGFR_L858R-Silybin_ | Leu718, Gly719, Phe723, Gly724, Thr725, Val726, Lys745, Glu746, Leu747, Leu788, Thr790, Phe795, Gly796, Cy797, Asp800, Tyr801, Leu844, Thr854, Leu1001 |
| EGFR_L858R-Gefitinib_ | Leu718, Gly719, Ala743, Ile744, Lys745, Met766, Leu788, Ile789, Thr790, Gln791, Leu792, Met793, Gly796, Cys797, Leu844, Thr854 |
| EGFR_T790M,L858R-Mangiferin_ | Leu718, Gly719, Ser720, Val726, Ala743, Thr790, Gln791, Leu792, Met793, Gly796, Cys797, Leu799, Arg841, Asn842, Leu844, Thr854 |
| EGFR_T790M,L858R-Silybin_ | Leu718, Gly719, Ser720, Gly721, Val726, Ala743, Lys745, Cys775, Met790, Gln791, Leu792, Met793, Cys797, Leu844, Thr854, Asp855, Leu1001 |
| EGFR_T790M,L858R-Gefitinib_ | Leu718, Gly719, Val726, Ala743, Ile744, Lys745, Met766, Leu788, Met790, Gln791, Leu792, Met793, Gly796, Cys797, Leu844, Thr854, Asp855, Phe997 |
| EGFR_G719S-Mangiferin_ | Leu718, Gly719, Val726, Tyr727, Ala743, Ile744, Lys745, Met766, Leu788, Ile789, Thr790, Gly796, Cys797, Arg841, Asn842, Leu844, Thr854, Asp855, |
| EGFR_G719S-Silybin_ | Leu718, Ser719, Val726, Phe795, Gly796, Cys797, Arg841, Asn842, Leu844, Thr854, Asp855, Leu1001 |
| EGFR_G719S-Gefitinib_ | Leu718, Gly719, Phe723, Val726, Ala743, Lys745, Met766, Leu777, Leu788, Thr790, Gln791, Leu792, Met793, Gly796, Cys797, Asp800, Leu844, Thr854, |
| HER2_-Mangiferin_ | Leu726, Gly727, Ser728, Gly732, Val734, Ala751, Lys753, Thr798, Gln799, Leu800, Met801, Gly804, Cys805, Leu807, Asp808, Arg849, Leu852 |
| HER2_-Silybin_ | Val734, Ala751, Ile752, Lys753, Met774, , Leu796, Val797, Thr798, Cys805, Arg849, Asn850, Leu852, Thr862, Asp863, |
| HER2_-Gefitinib_ | Leu726, Gly727, Val734, Ala751, Ile752, Lys753, Met774, Leu785, Leu796, Val797, Thr798, Gln799, Leu800, Met801, Gly804, Cys805, Leu807, Asp808, V851, Leu852, Thr862, Asp863, Leu866 |
| HER2_-T798M-Mangiferin_ | Leu726, Gly727, Ser728, Gly729, Gly732, Thr733, Val734, Ala751, Lys753, Thr798, Gln799, Leu800, Met801, Tyr803, Gly804, Cy805, Arg849, Leu852 |
| HER2_-T798M-Silybin_ | Leu726, Gly727, Ser728, Gly729, Ala730, Phe731, Cys805, Arg849, Asn850, Asp863, Leu866, Lys883, Val884, Pro885, Trp888 |
| HER2_-T798M-Gefitinib_ | Leu726, Gly727, Ser728, Gly729, Thr733, Val734, Ala751, Leu800, Gly804, Cys805, Arg849, Asn850, Leu852, Thr862, Asp863 |

**Table S2**. Per-residue energy decomposition for EGFR-ligand complexes after 100 ns-long MD simulations (kcal/mol).

| Residue | EGFR-_Mangiferin_ | EGFR-_Silybin_ | EGFR-_Gefitinib_ |
| --- | --- | --- | --- |
| Leu718 | -2.159 | -2.30133 | -1.815 |
| Gly719 |  | -0.23733 | -0.45433 |
| Phe723 |  | -0.207 |  |
| Val726 | -2.00167 | -1.429 | -1.748 |
| Ala743 | -2.08133 | -0.422 | -1.26233 |
| Ile744 | -0.69933 |  | -0.62267 |
| Lys745 | -1.81167 | -0.410 | -0.73633 |
| Leu777 | -0.47533 |  | -0.373 |
| Leu788 |  |  | -0.79433 |
| Ile789 | -0.77367 |  | -0.53633 |
| Thr790 | -0.97733 | -0.248 | -1.26867 |
| Gln791 | -0.384 | -0.385 | -0.72967 |
| Leu792 | -0.99867 | -0.84433 | -1.802 |
| Met793 | -0.26433 | -0.70367 | -1.34833 |
| Phe795 |  | -0.329 |  |
| Gly796 | -0.80933 | -1.787 | -0.89033 |
| Cys797 | -1.26633 | -1.80933 | -1.05167 |
| Asp800 | -1.315 | -0.427 |  |
| Leu844 | -1.932 | -0.99933 | -2.17067 |
| Thr854 | -1.47733 | -0.308 | -0.66633 |
| Asp855 | -1.29367 |  |  |
| Phe997 |  | -1.108 |  |
| Leu1001 | -0.85867 | -1.17 | -0.426 |

**Table S3**. Per-residue energy decomposition for EGFR_L858R_-ligand complexes after 100 ns-long MD simulations (kcal/mol).

| Residue | EGFR_L858R-Mangiferin_ | EGFR_L858R-Silybin_ | EGFR_L858R-Gefitinib_ |
| --- | --- | --- | --- |
| Leu718 | -1.87367 | -1.95767 | -1.941 |
| Gly719 | -0.531 | -0.557 | -0.31733 |
| Phe723 |  | -1.332 |  |
| Gly724 |  | -0.409 |  |
| Thr725 |  | -0.51767 |  |
| Val726 | -2.35633 | -2.49033 |  |
| Ala743 | -1.02367 |  | -1.21833 |
| Ile744 | -1.28233 |  | -0.65633 |
| Lys745 | -1.734 | -1.9405 | -0.85 |
| Glu746 |  | -0.295 |  |
| Leu747 |  | -0.8495 |  |
| Glu762 | -1.01333 |  |  |
| Met766 | -0.58767 |  | 0.66133 |
| Leu788 | -0.81733 | -0.1575 | -0.72333 |
| Ile789 |  |  | -0.44367 |
| Thr790 | -0.976 | -0.198 | -1.14833 |
| Gln791 |  |  | -0.29333 |
| Leu792 |  |  | -1.705 |
| Met793 |  |  | -2.09933 |
| Phe795 |  | -1.07 |  |
| Gly796 | -1.203 | -0.68467 | -0.97467 |
| Cys797 | -2.41433 | -1.97833 | -0.656 |
| Asp800 |  | -0.7755 |  |
| Tyr801 |  | -0.748 |  |
| Arg841 | -1.480 |  |  |
| Asn842 | -0.32967 |  |  |
| Leu844 | -1.91933 | -1.393 | -2.08667 |
| Thr854 | -1.22767 | -0.33767 | -0.688 |
| Asp855 | -0.54033 |  |  |
| Leu1001 |  | -1.38 |  |

**Table S4**. Per-residue energy decomposition for EGFRG_T790M-L858R_-ligand complexes after 100 ns-long MD simulations (kcal/mol).

| Residue | EGFRG_T790M-L858R-Mangiferin_ | EGFRG_T790M-L858R-Silybin_ | EGFRG_T790M-L858R-Gefitinib_ |
| --- | --- | --- | --- |
| Leu718 | -2.40667 | -1.20467 | -1.603 |
| Gly719 | -1.49833 | -1.616 | -0.44567 |
| Ser720 | -0.62833 | -1.64067 |  |
| Gly721 |  | -0.276 |  |
| Val726 | -1.507 | -1.61133 | -1.71867 |
| Ala743 | -0.741 | -0.86367 | -1.39367 |
| Ile744 |  |  | -0.529 |
| Lys745 |  | -1.71267 | -1.26367 |
| Met766 |  |  | -0.23033 |
| Cys775 |  | -0.351 |  |
| Leu788 |  |  | -0.95633 |
| Met790 | -0.69633 | -0.85667 | -2.08467 |
| Gln791 | -0.36533 | -0.77167 | -0.27267 |
| Leu792 | -1.11533 | -1.348 | -1.74467 |
| Met793 | -0.84733 | -1.25467 | -1.847 |
| Gly796 | -0.65167 |  | -1.02233 |
| Cys797 | -1.55633 | -0.63967 | -1.413 |
| Leu799 | -0.61767 |  |  |
| Arg841 | -1.319 |  |  |
| Asn842 | -0.454 |  |  |
| Leu844 | -1.70867 | -2.01367 | -2.21067 |
| Thr854 | -0.418 | -1.01167 | -0.84367 |
| Asp855 |  | -0.488 | -0.549 |
| Leu1001 |  | -0.472 |  |
| Phe997 |  |  | -0.6395 |

**Table S5**. Per-residue energy decomposition for EGFRG_G719S-_-ligand complexes after 100 ns-long MD simulations (kcal/mol).

| Residue | EGFRG_719S-Mangiferin_ | EGFRG_719S-Silybin_ | EGFRG_719S-Gefitinib_ |
| --- | --- | --- | --- |
| Leu718 | -2.163 | -2.163 | -1.91933 |
| Ser719 | -0.57967 | -0.57967 | -0.42333 |
| Phe723 |  |  | -0.116 |
| Val726 | -2.38633 | -2.38633 | -1.64367 |
| Tyr727 | -0.21167 |  |  |
| Ala743 | -0.70133 |  | -1.216 |
| Ile744 | -0.83333 |  |  |
| Lys745 | -1.17867 |  | -0.82533 |
| Met766 | -0.231 |  | -0.744 |
| Leu777 |  |  | -0.254 |
| Leu788 | -0.377 |  | -0.759 |
| Ile789 | -0.296 |  |  |
| Thr790 | -0.568 |  | -1.177 |
| Gln791 |  |  | -0.32967 |
| Leu792 |  |  | -1.67833 |
| Met793 |  |  | -2.23267 |
| Phe795 |  | -0.297 |  |
| Gly796 | -1.2 | -1.2 | -0.95067 |
| Cys797 | -1.94033 | -1.94033 | -0.717 |
| Asp800 |  |  | -0.259 |
| Arg841 | -1.11 | -1.119 |  |
| Asn842 | -0.22 | -0.23 |  |
| Leu844 | -1.65667 | -1.65667 | -2.18933 |
| Thr854 | -0.800 | -0.800 | -0.69767 |
| Asp855 | -0.311 | -0.311 |  |
| Leu1001 |  | -0.138 |  |

**Table S6**. Per-residue energy decomposition for HER2_-_-ligand complexes after 100 ns-long MD simulations (kcal/mol).

| Residue | HER2-_Mangiferin_ | HER2-_Silybin_ | HER2-_Gefitinib_ |
| --- | --- | --- | --- |
| Leu726 | -2.028 |  | -1.718 |
| Gly727 | -1.26467 |  | -0.53167 |
| Ser728 | -0.62233 |  |  |
| Gly732 | -0.4805 |  |  |
| Val734 | -2.26067 | -1.98233 | -1.70667 |
| Ala751 | -0.754 | -1.24167 | -1.33933 |
| Ile752 |  | -1.085 | -0.51867 |
| Lys753 | -1.324 | -2.06067 | -0.97367 |
| Met774 |  | -0.98067 | -0.38 |
| Leu785 |  |  | -0.384 |
| Leu796 |  | -2.25433 | -0.916 |
| Val797 |  | -0.81633 | -0.31933 |
| Thr798 | -0.27567 | -1.21267 | -1.18633 |
| Gln799 | -0.542 |  | -0.28367 |
| Leu800 | -1.54 |  | -1.776 |
| Met801 | -1.461 |  | -1.94867 |
| Gly804 | -0.9145 |  | -1.10567 |
| Cys805 | -2.02767 | -0.406 | -0.978 |
| Leu807 | -0.97367 |  | -0.14167 |
| Asp808 | -0.438 |  | -0.4315 |
| Arg849 | -0.681 | -1.866 |  |
| Asn850 |  | -0.683 |  |
| Val851 |  |  | -0.139 |
| Leu852 | -1.70567 | -1.045 | -2.184 |
| Thr862 |  | -1.50433 | -0.67467 |
| Asp863 |  | -0.6825 |  |
| Leu866 |  |  | -0.221 |

**Table S7**. Per-residue energy decomposition for HER2_T798_-ligand complexes after 100 ns-long MD simulations (kcal/mol).

| Residue | HER2_T798M-Mangiferin_ | HER2_T798M-Silybin_ | HER2_T798M-Gefitinib_ |
| --- | --- | --- | --- |
| Leu726 | -1.76267 | -1.79967 | -2.32767 |
| Gly727 | -1.09767 | -1.214 | -0.695 |
| Ser728 | -0.74467 | -0.88733 | -0.5725 |
| Gly729 | -0.79967 | -1.208 | -0.2355 |
| Ala730 |  | -0.76067 |  |
| Phe731 |  | -1.35233 |  |
| Gly732 | -0.429 |  |  |
| Thr733 | -0.619 |  | -0.446 |
| Val734 | -2.61167 |  | -1.767 |
| Ala751 | -0.94967 |  | -0.69733 |
| Lys753 | -2.10233 |  |  |
| Thr798 | -1.262 |  |  |
| Gln799 | -0.58133 |  |  |
| Leu800 | -1.36133 |  | -1.15267 |
| Met801 | -1.07167 |  |  |
| Tyr803 | -0.147 |  |  |
| Gly804 | -0.47867 |  | -0.92 |
| Cys805 | -1.25867 | -0.97233 | -1.69833 |
| Arg849 | -1.716 | -1.66933 | -1.262 |
| Asn850 |  | -0.1805 | -0.64 |
| Leu852 | -1.70633 |  | -1.46067 |
| Thr862 |  |  | -0.621 |
| Asp863 |  | -0.62367 | -0.20367 |
| Leu866 |  | -0.40533 |  |
| Lys883 |  | -0.335 |  |
| Val884 |  | -0.366 |  |
| Pro885 |  | -0.628 |  |
| Trp888 |  | -0.3135 |  |
